# Barriers and facilitators for community pharmacists’ participation in pharmacy practice research: a survey

**DOI:** 10.1111/ijpp.12522

**Published:** 2019-02-19

**Authors:** Esther Kuipers, Michel Wensing, Peter A.G.M. De Smet, Martina Teichert

**Affiliations:** ^1^ Department of IQ Healthcare Radboud Institute for Health Sciences Radboud University Medical Centre Nijmegen The Netherlands; ^2^ BENU Apotheek Zeist West Zeist The Netherlands; ^3^ Department of General Practice and Health Services Research University Hospital Heidelberg Heidelberg Germany; ^4^ Department of Clinical Pharmacy Radboud Institute for Health Sciences Radboud University Medical Centre Nijmegen The Netherlands; ^5^ Department of Clinical Pharmacy & Toxicology Leiden University Medical Centre Leiden The Netherlands

**Keywords:** community pharmacy, pharmacist opinions, pharmacy practice research, survey, the Netherlands

## Abstract

**Objectives:**

The aim of this study was to explore pharmacists’ barriers and facilitators regarding participation in pharmacy practice research.

**Methods:**

We conducted an online cross‐sectional survey in 1974 community pharmacies in the Netherlands.

**Key findings:**

A total of 252 pharmacists completed the questionnaire. The majority agreed that participation in research should be part of daily practice. Efficient time investment and a clear benefit for general professional knowledge, patient care and pharmacy organisation were the most important facilitating factors.

**Conclusions:**

To encourage pharmacists’ participation, researchers should offer clear instructions, possibilities for flexible time management, simple patient inclusion, task delegation and no additional contacts with healthcare professionals due to the research.

## Introduction

Effective primary care, based on the relationship between healthcare professional and patient, requires practice research to examine the need, effectiveness and efficiency of specific services that will provide evidence to inform best practices.^1^, ^2^ In the past decades, community pharmacy practice has extended the traditional role of dispensing medication to one including provision of patient‐centred pharmaceutical care.^2^, ^3^ However, conducting practice research and recruiting healthcare professionals to participate in the practice can be challenging. It is essential to accurately grasp pharmacists’ views and potential barriers, and prevent dropouts during studies.^4^ Previous studies have not specifically targeted community pharmacists, and the study results may be outdated.^2^, ^5^, ^6^, ^7^

The present study aimed to identify community pharmacists’ barriers and facilitators in considering participation in pharmacy practice research in the Netherlands. It did so by describing these pharmacists’ views and attitudes so as to provide researchers with insight on how to optimise research participation.

## Method

### Setting

Pharmacy students and community pharmacists in the Netherlands are trained to perform research and are regularly invited to participate in pharmacy practice research.

### Survey

A cross‐sectional survey was performed in 2017. All pharmacists from 1974 community pharmacies in the Netherlands^8^ were invited to complete a 35‐item questionnaire. Statements regarding views and attitudes were scored on a 10‐point Likert scale. Statements were based on literature^2^, ^5^, ^6^, ^7^ and researchers’ experiences, and all of the present study's authors checked them for face validity.

A specific example of a pharmacy practice study was also developed to explore the willingness to participate by varying the (extent of) different potential barriers. The questionnaire ended with an open‐ended question on key factors for participation.

No personal identifiers were collected.

### Data collection

An e‐mail invitation to participate in the survey was sent in July 2017. Non‐responders were sent a reminder 1 week later. Data collection were completed at the end of the same month.

### Data analysis

Quantitative data were analysed using descriptive statistics. For each statement, the median and interquartile range were calculated, together with numbers of pharmacists who noted scores of 1 or 2 and 9 or 10. Two of the authors (EK and MT) independently coded and summarised qualitative responses to the open‐ended question to identify key topics via the grounded theory approach.^9^ Disagreements were discussed until consensus was reached.

## Results

The questionnaire was completed by 252 of the 2968 contacted pharmacists; response rate: 8.8%. Their mean age was 43.7 years, and nearly 48% had recently participated in practice research (within the preceding year).

Table 1 shows the pharmacists’ general views and attitudes regarding pharmacy practice research. A majority (85.6%) agreed (score: 7–10 points) that practice research provided evidence‐based insight into the activities of community pharmacists and opportunities for professional development. A majority (71%) also agreed that participation in practice research in general is a natural part of the pharmacists’ profession.

**Table 1:** Pharmacists’ views and attitudes regarding practice research

Statement	Median (IQR)	Number of respondents score 1 or 2^a^	Number of respondent score 9 or 10	Agree (%)^b^
Participation in pharmacy practice research belongs to the profession of every community pharmacist	8 (2)	13	57	179 (71.0%)
Participation in pharmacy practice research belongs to the education of a community pharmacy specialist	8 (2)	10	88	220 (87.3%)
Without pharmacy practice research the specialism of the community pharmacist cannot exist for the long term	7 (2)	17	54	162 (64.3%)
Pharmacy practice research provides evidence‐based insights into the actions of the community pharmacist	8 (2)	7	83	216 (85.7%)
Pharmacy practice research provides insights into future opportunities for the profession of the community pharmacist	8 (2)	4	75	209 (82.9%)
I would like to participate in pharmacy practice research, but it is too busy in the pharmacy	7 (3)	28	47	139 (55.2%)
I only participate in pharmacy practice research if the subject is interesting enough for me	8 (1)	8	55	198 (78.6%)
If the subject is also important for the general practitioners (GP's) I am working with, I only participate if patients’ GP has no objections	7 (4)	31	36	127 (50.4%)
I am willing to find time to participate in pharmacy practice research	7 (3)	20	25	130 (51.6%)
I only participate in pharmacy practice research if have confidence in the investigators	8 (2)	5	73	190 (75.4%)
I only participate in pharmacy practice research if it is obliged (e.g. during education)	4 (4)	80	6	38 (15.1%)
If I participate in pharmacy practice research depends on my employer	5 (5.75)	73	25	84 (33.3%)
I only participate in pharmacy practice research if I know the investigators personally	3 (3)	124	0	6 (2.4%)
I am convinced of the added value of pharmacy practice research	8 (2)	4	65	192 (76.2%)
Participating in pharmacy practice research…				
Is generally interesting for me	7 (2)	13	49	188 (74.6%)
Gives me opportunities for personal development as a pharmacist	8 (2)	11	46	183 (72.6%)
Is feasible in the pharmacy where I am working	7 (3)	22	28	128 (50.8%)
Is usual procedure in the pharmacy where I am working	5 (3)	63	17	73 (29%)
Is stimulated by colleagues or the professional group	6 (3)	29	12	83 (32.9%)
Can help me to improve patient care and my relation with patients	7 (2)	6	29	167 (66.3%)
Can help me to improve my position as healthcare professional	8 (2)	4	65	202 (80.2%)

IQR, interquartile range.

^a^Total number of respondents per statement: 252. ^b^Scores 7–10.

Almost 51% of the respondents felt participation was feasible in daily practice, and 29% regarded participation as common for their daily practice. Additionally, 55% indicated they would like to participate but lacked the time to do so. Important facilitators were confidence in the investigators and interest in the study topic (75.4% and 78.6% agreement, respectively). Although a majority (76%) of the respondents reported being convinced of the general added value of pharmacy practice research, only 52% indicated they were prepared to invest the time to participate.

The surveyed pharmacists expressed they were more likely to participate in practice research when the requested work could be spread over some weeks rather than performed in 1 day (77.4% versus 35.3%, respectively; Table 2). They also preferred the possibility of delegating tasks (e.g. to their pharmacy technicians) over performing all procedures themselves (90.9% versus 61.9%, respectively). They were more likely to participate when patients could be invited by email (86.8%) instead of personally during pharmacy visits or by telephone (65.9%). Pharmacists can access patients’ email addresses, as most pharmacies offer digital services (e.g. track and trace). The need to cooperate with medical specialists discouraged more than half of the surveyed pharmacists; only 48% would participate, compared with 73.8% when cooperation only with general practitioners was required.

**Table 2:** Case: organisational factors and influence on participation (*n* = 252)

You are invited to participate in a pharmacy practice study about the implementation of specific clinical rules. You have to include five patients, obtain their informed consent and collect data as well by a short questionnaire as from the pharmacy information system. The anonymous data have to be registered in a predefined form and should be sent digitally to the investigators. The estimated total time investment is about 5 hours	
*Would you participate in the described study if:*	*Yes (%)*
The total investment of time has to be done on 1 day?	89 (35.3)
The total investment of time has to be spread over a period of 3 weeks?	195 (77.4)
The study has to be finished within 4 weeks from now?	169 (67.1)
The study has to be finished within 12 weeks from now?	200 (79.4)
All tasks have to be done by the pharmacist?	156 (61.9)
You can delegate a part of the tasks?	229 (90.9)
There is no financial compensation?	159 (63.1)
During the study cooperation with the general practitioner is required?	186 (73.8)
During the study cooperation with a medical specialist is required?	121 (48.0)
You can invite all patients for participation directly in the pharmacy (no selection needed)?	211 (83.7)
You have to make a selection before inviting patients yourself?	203 (80.6)
You can invite patients for participation by email?	219 (86.9)
You can invite patients for participation only personally or by telephone?	166 (65.9)

A total of 415 key factors for participation were mentioned. The cluster of factors identified most frequently were total time investment, timing of the study and the possibility of flexible time needed to participate (*n *= 142). These were followed by need for clear added value (for the profession, the patient and the pharmacy practice or pharmacist, *n *= 104). Specifically mentioned positive factors (*n *= 67) were simple patient selection and data collection, no need for cooperation with many different healthcare professionals, a clear and complete description of the required tasks, no collection of superfluous data, and reliable explanation of the study and the activities required.

## Discussion

The present study results offer clear guidance for designing pharmacy practice studies. Researchers should pay close attention to efficient time investment and study logistics, for example possibilities of inviting patients by email, delegating tasks and spreading out time investment. This study corroborates results of earlier studies.^2^, ^10^ When pharmacists were convinced of a study's added value and feasibility, they reported willingness to invest their time, even when no financial compensation was available. However, obligated contact and cooperation with other healthcare professionals was a discouraging factor in participating in practice research.

A limitation in the present survey was the low‐response rate, which reflected the problem addressed herein. As expected, respondents had recently participated in pharmacy practice research. The results cannot be generalised to all community pharmacists because of this selection bias. However, the opinions of experienced and interested respondents were of great importance. Committed pharmacists experienced limited support from colleagues or professional organisations; thus, there are still possibilities to actively boost participation.

## Conclusion

Pharmacists’ participation in practice research depends on the research design. Clear descriptions, possibilities for flexible time management, simple patient inclusion and task delegation can all increase this participation. Researchers should acknowledge that cooperation with many different healthcare professionals may pose a barrier towards participation in practice research and should develop strategies to address this.

## Declarations

### Conflict of interest

The Author(s) declare(s) that they have no conflicts of interest to disclose.

### Funding

This work was supported by unconditional research grants from the Royal Dutch Association for the advancement of pharmacy (KNMP) (grant number PR16_1410), and AstraZeneca (grant number 2603949255).

### Authors’ contributions

EK, PDS, MW and MT designed the study. EK collected the data. EK and MT analysed the data. EK wrote the draft of the manuscript. EK, PDS, MW and MT interpreted the findings and co‐edited the paper. All Authors state that they had complete access to the study data that support the publication.
